# Structure Design on Thermoplastic Composites Considering Forming Effects

**DOI:** 10.3390/polym16202905

**Published:** 2024-10-15

**Authors:** Wei Xie, Kai Song, Ju Yang, Fengyu Wang, Linjie Dong, Shengjie Jin, Guohua Zhu, Zhen Wang

**Affiliations:** 1 State Key Laboratory of Advanced Design and Manufacturing Technology for Vehicle, Hunan University, Changsha 410082, China; wxiel@jmev.com; 2 School of Automobile, Chang’an University, Xi’an 710064, China; 18042246560@163.com (J.Y.); wangfengyu0429@163.com (F.W.); dhe300205@163.com (L.D.); 15053310985@163.com (S.J.); guohuazhu@chd.edu.cn (G.Z.)

**Keywords:** thermoplastic composites, stamping process, bending performance, stamping–bending coupled model, multi-objective discrete optimization

## Abstract

Carbon fiber reinforced polypropylene (CF/PP) thermoplastics integrate the superior formability of fabrics with the recoverable characteristics of polypropylene, making them a pivotal solution for achieving lightweight designs in new energy vehicles. However, the prevailing methodologies for designing the structural performance of CF/PP vehicular components often omit the constraints imposed by the manufacturing process, thereby compromising product quality and reliability. This research presents a novel approach for developing a stamping–bending coupled finite element model (FEM) utilizing ABAQUS/Explicit. Initially, the hot stamping simulation is implemented, followed by the transmission of stamping information, including fiber yarn orientation and fiber yarn angle, to the follow-up step for updating the material properties of the cured specimen. Then, the structural performance analysis is conducted, accounting for the stamping effects. Furthermore, the parametric study reveals that the shape and length of the blank holding ring exerted minimal influence on the maximum fiber angle characteristic. However, it is noted that the energy absorption and crushing force efficiency metrics of the CF/PP specimens can be enhanced by increasing the length of the blank holding ring. Finally, a discrete optimization design is implemented to enhance the bending performance of the CF/PP specimen, accounting for the constraint of the maximum shear angle resulting from the stamping process. The optimized design resulted in a mass reduction of 14.3% and an improvement in specific energy absorption (*SEA*) by 17.5% compared to the baseline sample.

## 1. Introduction

The majority of conventional thin-walled metallic automobile body components, such as frontal rails, side rails and external/internal door panels, are predominantly manufactured through the stamping process. This process inevitably generates various typical stamping characteristics, including material thinning, wrinkle deformation and residual stress/strain [1,2]. These stamping characteristics will subsequently be transmitted to the formed structure and further affect the product performance, which is referred to as the stamping effect. How to comprehensively account for stamping effects in the structural design procedure has emerged as a significant technical challenge for the automobile industry in recent years. To address this critical issue, the innovative concept of process–performance coupled design has gained recognition and traction within the automobile industry, in which process refers to stamping or manufacturing process, and performance pertains to the structural efficacy of the components. Encouragingly, the process–performance coupled design methodology for metallic vehicular components has seen substantial advancements in theoretical, numerical and optimization models owing to the persistent efforts of numerous scholars. This progress has significantly facilitated the widespread application of metallic materials in the automotive field [3,4,5].

Woven fabric reinforced thermoplastic (WFRTP), as an advanced composite material with lightweight, high strength and environmentally friendly advantages, has exhibited broad application prospects in the automotive lightweight field [6,7,8,9,10]. Similar to the manufacturing process of sheet metals, the hot stamping process will result in alterations in resin content, variations in fiber yarn angles, thickness reductions and the generations of residual stress and strain within WFRTP laminates [11,12,13,14,15]. Thus, it is necessary to take stamping effects into account for further structural performance design of WFRTP components. Despite the process–performance coupled design methodology has been effectively implemented in metallic thin-walled structures [1,2,3,4,5], however, the stamping mechanisms, material constitutive, design variables and optimization objectives of WFRTPs are quite different from those of metallic materials [16,17,18,19,20]. This disparity renders the existing process–performance coupled design methodologies for sheet metals inadequate for directly guiding the design of WFRTP structures. There is a notable lack of literature addressing the process–performance coupled design methodology specifically for WFRTP structures.

The establishment of an efficient and accurate process–performance coupled model represents a critical challenge in achieving integrated optimization design for the WFRTP vehicular components. Recently, a few scholars have contributed numerous efforts to address this significant issue and yielded valuable insights [21,22,23,24,25,26,27]. Kärger et al. [21] introduced a comprehensive computer-aided engineering (CAE) design chain aiming at facilitating the integrated optimization design of continuous fiber-reinforced composite components, which comprehensively considered the forming, compacting, curing, mapping and compressing processes. Han et al. [25] established the stamping–compressing coupled finite element model (FEM) to predict compressive failure behaviors of thermosetting composite structures accounting for the effects of fiber yarn angle variations. These studies indicate that the development of a reliable process–performance coupled FEM for WFRTP structures is fundamentally linked to the accuracy of the stamping FEM, crashing FEM and process information mapping algorithm. Furthermore, additional processes such as cutting, trimming and assembly should also be integrated into the coupled FEM to ensure that simulations closely reflect real-life design processes. Previous studies mainly focused on the coupled FEM for the thermosetting composites, and there are few reports addressing the establishment of such a coupled FEM for WFRTPs.

How to implement an efficient optimization design based on the process–performance coupled model to fully exploit the potential of materials is another critical challenge in the integrated design of WFRTP structures. Analyzing the integrated design characteristics of energy-absorbing WFRTP structures reveals that adherence to manufacturing requirements is a fundamental prerequisite for the subsequent performance design of cured structures [1,2,3,5,21]. In other words, formability can be conceptualized as the manufacturing constraint within the structural optimization design. Therefore, the core objective of the integrated design of the WFRTP vehicular structure is to determine the optimum metrics from a vast array of design variable combinations (including stamping parameters, material variables and structure geometric dimensions, etc.) with constraints of the forming process. Kärger et al. [28] studied the forming-stiffness integrated optimization design for the composite beam through a two-loop optimization approach and yielded favorable outcomes, but the nonlinear large deformation behaviors and failure models of materials have not been considered. There is still a lack of reports on the process–performance coupled optimization design for thin-walled energy-absorbing WFRTP structures.

The existing process–performance coupled finite element method (FEM) for thermosetting composite structures primarily involves three essential steps: conducting the forming analysis using ABAQUS or LS-DYNA; mapping the process information through MATLAB or PYTHON; and evaluating the structural performance again in ABAQUS or LS-DYNA. While these coupled numerical models effectively account for the influence of the forming process on structural performance, the calculation procedure is inherently discontinuous due to the mapping process not being integrated within the finite element analysis tool itself. This disjunction may hinder the design efficiency of composite structures. On the other hand, thermoplastic composites exhibit more pronounced plastic characteristics compared to their thermosetting counterparts, rendering the elastic–brittle constitutive model employed for structural performance analysis of thermosetting composite inappropriate for thermoplastic composites. Therefore, it is imperative to continue advancing the numerical modeling and design methodologies specifically tailored for thermoplastic composites.

This study explores the structural design methodology for carbon fiber reinforced polypropylene (CF/PP) thermoplastic composite vehicular components accounting for stamping process effects. First, the bias-extension test was performed on a single CF/PP prepreg at a temperature of 220 °C to provide parameters for the subsequent numerical model of the stamping process. The shear mechanical parameters of CF/PP laminates with three different fiber angles were further obtained by bias-extension tests to provide parameter supports for the bending numerical model. The six-layer hat-shaped CF/PP tube with a stacking sequence of (+45°/−45°)_6_ was manufactured by the hot stamping process and subsequently subjected to the quasi-static three-point bending (TPB) test. Then, the stamping–bending coupled constitutive model and finite element model (FEM) was developed and validated, and the effects of the mold cross-sectional shape and the blank holding ring shape/length on forming and bending performances of the CF/PP tube were discussed. Finally, the multi-objective optimization design for the CF/PP tube was conducted. The present work introduces an innovative process–performance coupled finite element method (FEM) specifically for thermoplastic composites. This method facilitates the automatic transmission of stamping information across various analysis steps while maintaining a continuous computation of the coupled models. Additionally, the multi-objective discrete optimization strategy offers an efficient design framework for thermoplastic composite structures.

## 2. Stamping–Bending Coupled FEM Development and Validation

### 2.1. Experimental Procedure for Stamping–Bending Process

**Figure 1:**
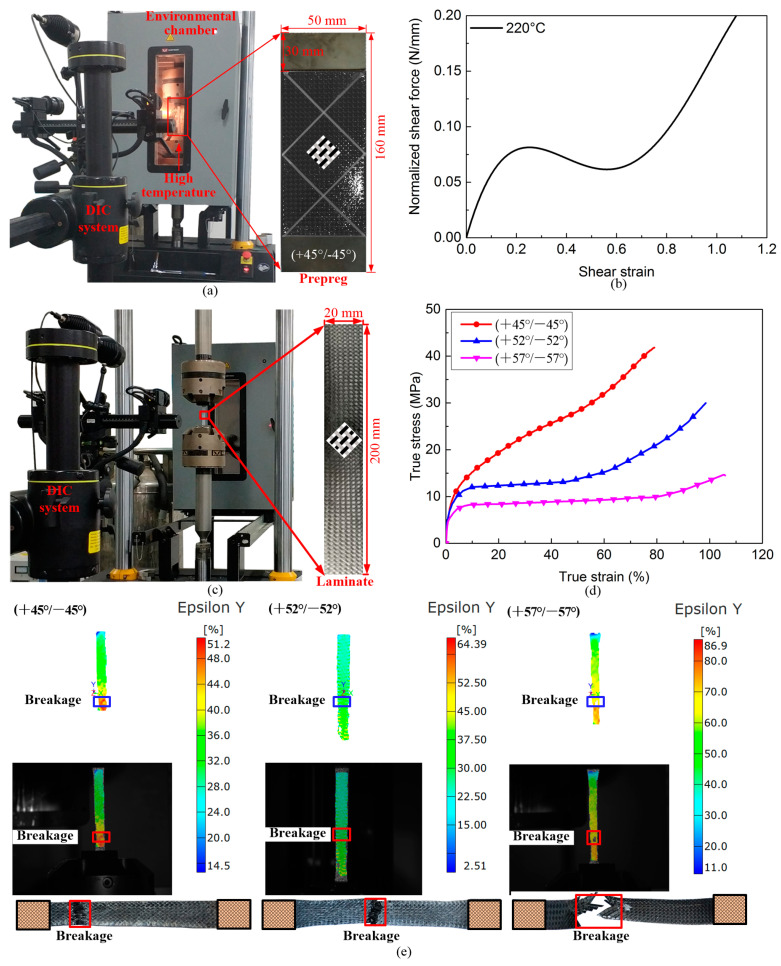
Mechanical property parameter characterizations of CF/PP prepregs and the CF/PP laminates: (**a**) the bias-extension equipment for the CF/PP prepreg; (**b**) the normalized shear force–shear strain curve of single CF/PP prepreg; (**c**) the bias-extension equipment for the CF/PP laminate; (**d**) the true stress–true strain curves of CF/PP laminates; and (**e**) failure positions of three different CF/PP tensile samples.

Since shear deformations predominantly influence the forming behavior of CF/PP prepregs in the stamping process, it is essential to obtain shear characterization data for the individual CF/PP prepreg under the relevant forming temperature condition. This information is critical for the development of an accurate finite element model for the stamping process. The bias-extension test and the picture-frame test are two established methodologies for characterizing the shear properties of both dry fabrics and pre-impregnated fabrics [29,30,31,32]. In the present study, the bias-extension technique was employed to characterize the normalized shear force–shear strain curve of CF/PP prepregs at the stamping temperature of 220 °C, as shown in Figure 1a,b. The normalized shear force ($F_{sh}$) can be calculated as follows:

(1) $$F_{sh}\left(\gamma \right)=\frac{1}{\left(2H-3W\right)\cos\gamma }\left(\left(\frac{H}{W}-1\right)\cdot F\cdot \left(\cos\frac{\gamma }{2}-\sin\frac{\gamma }{2}\right)-W\cdot F_{sh}\left(\frac{\gamma }{2}\right)\cdot \cos\frac{\gamma }{2}\right)$$

where H and W denote the length and the width of the specimen, respectively, F represents the tensile force and γ is the shear angle.

In the pre-heating stage, all CF/PP prepregs were oriented at an angle of 45°. However, the fiber direction and fiber angle (i.e., the angle between the weft and warp yarns) will change due to the constraint of the blank holding ring in the forming stage. Moreover, the cured hat-shaped specimen was subsequently subjected to the three-point bending (TPB) test, where the off-axis properties of the cured laminate significantly affect the bending behavior of the hat-shaped specimen. Consequently, it is imperative to evaluate the shear properties of CF/PP laminates with varying fiber angles to develop a reliable finite element model for the three-point bending analysis. For this purpose, three different laminates with fiber angles—(+45°/−45°), (+52°/−52°) and (+57°/−57°)—were prepared, as shown in Figure 1c,d. According to Figure 1d, it can be seen that the fiber breakage and matrix cracks dominated the failure process of all laminates, and their longitudinal failure strain increased with increasing fiber yarn angle. The (+57°/−57°) sample exhibited the highest longitudinal strain value due to the significant shear deformations.

**Figure 2:**
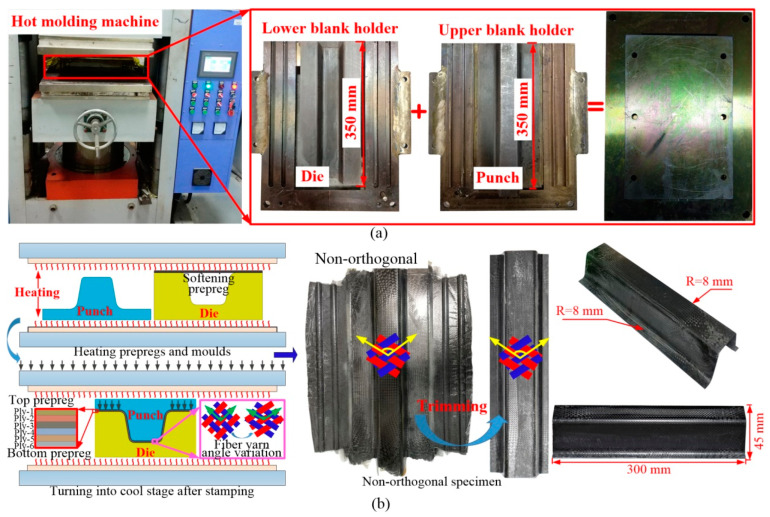
Manufacturing process of the hat-shaped CF/PP specimen: (**a**) the hot stamping machine and the molds; (**b**) the stamping molds and prepregs are pre-heated first, then the punch moves downwards and finishes the stamping step; the non-orthogonal cured specimen is taken out after cooling stage and trimmed to the final configuration for the further three-point bending test, the red line represents the weft yarn, and the blue line denotes the warp yarn, and the yellow arrow is the fiber direction.

Figure 2 illustrates the hot stamping process and the geometric dimensions of the hat-shaped CF/PP specimen. The stamping molds include the punch, the die and the blank holders, as shown in Figure 2a. Six layers of CF/PP prepregs with a stacking sequence of (+45°/−45°)_6_ were first pre-heated at a hot pressing machine at a temperature of 220 °C. Next, the hot pressing machine was opened, and the molds and prepregs were kept heated while the blank holders were quickly clamped tightly using six bolts. The pre-heated punch was then quickly positioned above the molten prepregs, and the hot pressing machine started to close, pressing the prepregs into the die and causing significant shear deformations in the fiber yarns, as shown in Figure 2b. Finally, the pre-fabricated specimen was removed from the machine after cooling to room temperature. The cured specimen was trimmed to its final configuration for the further three-point bending test, with the fiber angles in the inter-laminar laminates changing from an orthogonal to a non-orthogonal frame. Its geometric dimensions are shown in Figure 2b.

**Figure 3:**
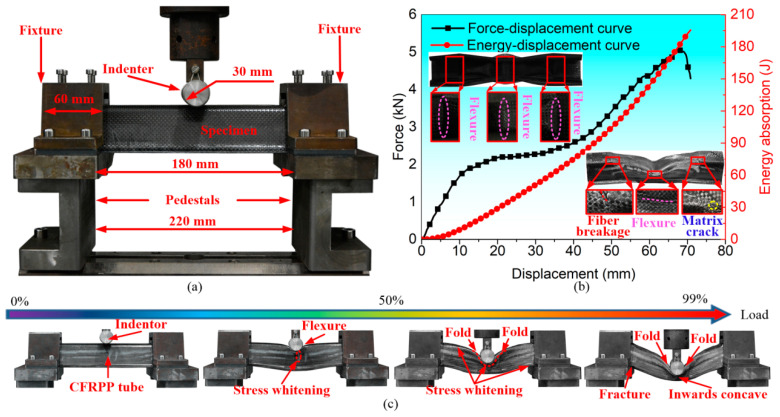
The three-point bending test fixtures and the typical bending responses of the CF/PP specimen: (**a**) three-point bending fixture descriptions; (**b**) force-/energy–displacement curves, deformation patterns and failure modes; and (**c**) typical historical photos of the CF/PP specimen.

Figure 3 illustrates the three-point bending test fixtures and experimental results of the six-layer hat-shaped CF/PP specimen. The trimmed CF/PP specimen was completely constrained by two hat-shaped clamps with a length of 60 mm, and the cylindrical indenter utilized in the experiment had a length of 150 mm and a diameter of 30 mm, as depicted in Figure 3a. The compressive velocity of the indenter remained constant at 4 mm/min, and the experiment was terminated when the compressive displacement reached 80 mm. The camera recorded the bending histories of the CF/PP specimen, and the test machine automatically recorded the compressive force and the moving length of the indenter. The compressive force quickly increased before the compressive displacement reached approximately 12 mm. Subsequently, the force increased at a significantly slower rate until the displacement reached about 45 mm, in which distinct flexural folds developed in the central region of the CF/PP specimen. Finally, the compressive force dropped sharply due to fiber breakage and matrix crack occurrences at a displacement of 69 mm, as depicted in Figure 3b,c.

### 2.2. Strategy for Stamping–Bending Coupled FEM Development

**Figure 4:**
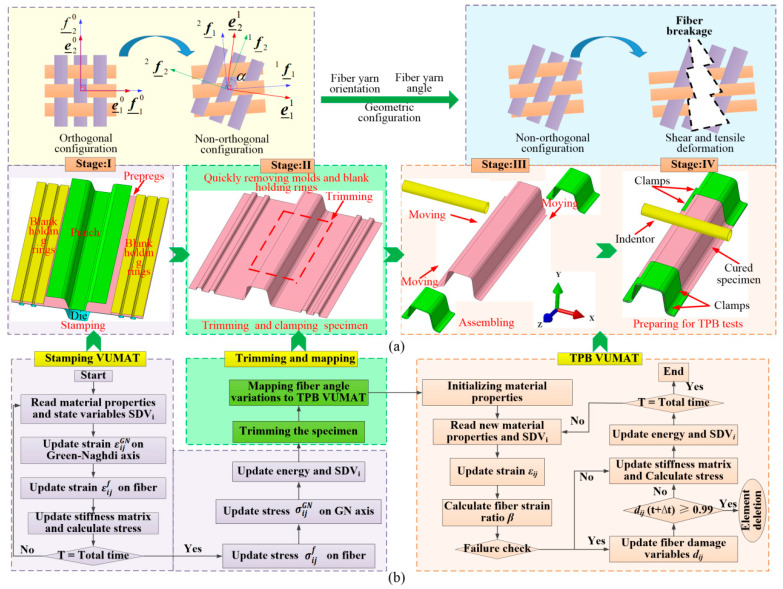
The establishment and workflow diagrams of the stamping–bending coupled model: (**a**) multi-step procedure of the stamping–bending coupled analysis in ABAQUS/Explicit, in which the orthogonal fabric configuration turned into a non-orthogonal configuration in the stamping step, and then the stamping information was transferred to the subsequent structural analysis step; (**b**) the VUMAT flowchart for the stamping–bending coupled finite element model, in which the trimming and mapping VUMAT served as a connecting link between the stamping VUMAT and bending VUMAT.

The key conception of stamping VUMAT involves the calculation of the fiber yarn angle and the updating of the stress tensor for CF/PP prepregs in each incremental step. Initially, the strain increments in the Green–Naghdi axis (GN-axis) are converted to the fiber axis, leading to updates in the fiber yarn angle and the corresponding stress increments of the fiber yarn. Subsequently, the fiber stress components are transformed back to the GN-axis again, as illustrated in Figure 4a. The comprehensive description of the computational algorithm and flowchart is available in prior publications [10,33,34,35,36,37], and several key steps will be elaborated upon in the subsequent sections.

In this study, a hypoelastic material law was utilized to effectively model the significant shear deformations of CF/PP prepregs during the stamping process, which can be articulated as follows:

(2) $${\underline{\underline{\sigma }}}^{\nabla }=\underline{\underline{\underline{\underline{C}}}}:\underline{\underline{D}}$$

where $\underline{\underline{\sigma }}$ is the stress tensor, $\underline{\underline{\underline{\underline{C}}}}$ denotes constitutive tensor and $\underline{\underline{D}}$ represents strain rate.

In ABAQUS software, the Green–Naghdi stress rate is employed to establish the mechanical constitutive relationship of materials within the VUMAT framework. The Cauchy stress tensor can be calculated as follows:

(3) $${\underline{\underline{\sigma }}}^{\nabla }=\underline{\underline{Q}}(\frac{d}{dt}({\underline{\underline{Q}}}^{T}\underline{\underline{\sigma }}\underline{\underline{Q}})){\underline{\underline{Q}}}^{T}$$

where $\underline{\underline{Q}}$ is the rotation tensor between the orthogonal frame and non-orthogonal frame.

It is worth noting that the strain increment of each integral point is initially specified along the GN-axis in each incremental step. Consequently, it must first be converted to the fiber frame as follows:

(4) $${\left[d\varepsilon \right]}_{fi}={\left[T_{i}\right]}^{T}{\left[d\varepsilon \right]}_{e}\left[T_{i}\right]$$

where $T_{i}$ is the transformation matrix, e and f denote the initial orthogonal frame and non-orthogonal fiber frame, respectively.

Then, the stress component of fiber yarn can be calculated as follows:

(5) $${\left[d\sigma \right]}_{fi}={\left[C_{i}\right]}_{fi}{\left[d\varepsilon \right]}_{fi}$$

where $C_{i}$ represents the elastic matrix.

Finally, the stress components along the fiber have to be converted to GN-axis:

(6) $${\left[\sigma \right]}_{e}={\left[T_{i}\right]\left[\sigma \right]}_{e}{\left[T_{i}\right]}^{T}$$

According to the results obtained from the three-point bending (TPB) test, the hat-shaped CF/PP hollow structure exhibited significant plastic deformations, culminating in fiber breakage as the failure mode occurred at the end of the test. The observed plastic behavior can be attributed to the off-axis loading conditions, wherein, additionally, the high ductility of the polypropylene matrix contributed to the progression of plastic deformations. Consequently, an elastic–plastic constitutive model was formulated to accurately represent the TPB behavior of the hat-shaped CF/PP hollow structure. The in-plane stress–strain relationship incorporated damage effects is expressed as follows [38,39,40]:

(7) $$\left\{\begin{array}{c} \varepsilon_{11}\\ \varepsilon_{22}\\ \varepsilon_{12}^{el} \end{array}\right\}=\left[\begin{array}{ccc} \frac{1}{\left(1-d_{ft1}\right)E_{1}} & \frac{{-\nu }_{12}}{E_{1}} & 0\\ \frac{{-\nu }_{21}}{E_{1}} & \frac{1}{\left(1-d_{ft2}\right)E_{2}} & 0\\ 0 & 0\; & \frac{1}{(1-d_{f12})2G_{12}} \end{array}\right]\left\{\begin{array}{c} \sigma_{11}\\ \sigma_{22}\\ \sigma_{12} \end{array}\right\}$$

where the ${\left\{\varepsilon_{11\;}\varepsilon_{22\;}\varepsilon_{12}^{el}\right\}}^{T}$ and ${\left\{\sigma_{11\;}\sigma_{22\;}\sigma_{12\;}\right\}}^{T}$ represent the strain and stress components, respectively. $d_{ft1}$, $d_{ft2}$ and $d_{f12}$ are damage factors.

Damage factors $d_{ft1}$, $d_{ft2}$ and $d_{f12}$ can be calculated as follows [41,42,43]:

(8) $$d_{fti}=\left\{\begin{array}{c} 1-e^{\frac{1}{mti}\left(1-{\left(\max\left(\frac{\varepsilon_{fti}}{\varepsilon_{FLC}}\right)\right)}^{mti}\right)},\;\;\left(\varepsilon_{fti}/\varepsilon_{FLC}\right)\geq 1\\ 0,\;\;\;\;\;\;\;\;\;\;\;\;\;\;\;\;\;\;\;\;\;\;\;\;\;\;\;\;\;\;\;\;\;\;\;\;\;\;\;\;\;\;\;\;\;\;\;\;\left(\varepsilon_{fti}/\varepsilon_{FLC}\right)<1 \end{array}\right.$$

(9) $$d_{f12}=\left\{\begin{array}{c} 1-e^{\frac{1}{m12}\left(1-{\left(\max\left(\frac{\varepsilon_{f12}}{\varepsilon_{12}}\right)\right)}^{m12}\right)},\;\;\left(\varepsilon_{f12}/\varepsilon_{12}\right)\geq 1\\ 0,\;\;\;\;\;\;\;\;\;\;\;\;\;\;\;\;\;\;\;\;\;\;\;\;\;\;\;\;\;\;\;\;\;\;\;\;\;\;\;\;\;\;\;\;\;\;\;\;\left(\varepsilon_{f12}/\varepsilon_{12}\right)<1 \end{array}\right.$$

(10) $$d_{fti}=\left\{\begin{array}{l} 0.03271+0.02571\beta ,\;\;-1\leq \beta \leq -0.3\\ 0.023,\;\;\;\;\;\;\;\;-0.3<\beta \leq 1 \end{array}\right.$$

where subscripts i represents 1 or 2; mti and m12 are the softening parameter for the stiffness reduction [42], and their values are 2.0 and 2.0, respectively; $\varepsilon_{fti}$ and $\varepsilon_{f12}$ denote the fiber strain and shear strain, respectively; and $\varepsilon_{FLC}$ is the equivalent fiber strain as reported in [43]. Shear failure strain $\varepsilon_{12}$ is detailed in Table 1. 

The shear plastic constitutive model used here is the bi-linear plastic model, as extensively detailed in reference [44]. In the plastic stage, the yield shear stress can be determined by the shear stress of the last incremental step (i.e., $\sigma_{n}^{Y}$), the slope of the plastic part of the bi-linear curve (i.e., K) and the current plastic strain increment (i.e., $\varepsilon_{n+1}^{pl}$), which is given as the following:

(11) $$\sigma_{n+1}^{Y}=\sigma_{n}^{Y}+K\Delta \varepsilon_{n+1}^{pl}$$

The bi-linear curves of CF/PP laminates with different fiber angles are shown in Figure 1d; the K values, yield strengths and shear parameters are listed in Table 1.

The interpolation method has been recognized as an efficient approach for transferring the stamping information, including thickness and residual stress from the stamping process to the crashing process in the stamping–crashing coupled analysis procedure of metal sheets [3,5]. Several researchers employed a similar method to develop the forming-performance coupled finite element models of composite sheets by integrating the ABAQUS or LS-DYNA with MATLAB and yielded favorable results [25,26]. This study introduces an innovative multi-step method to facilitate the stamping–bending coupled analysis procedure of the hat-shaped CF/PP specimen exclusively within ABAQUS. This method not only simplifies the stamping information mapping process but also improves the mapping accuracy by enabling the automatic and seamless transfer of forming information among multiple steps within ABAQUS. The stamping–bending coupled VUMAT and the analysis flowchart are depicted in Figure 4b.

### 2.3. Stamping–Bending Coupled FEM and Validation

**Figure 5:**
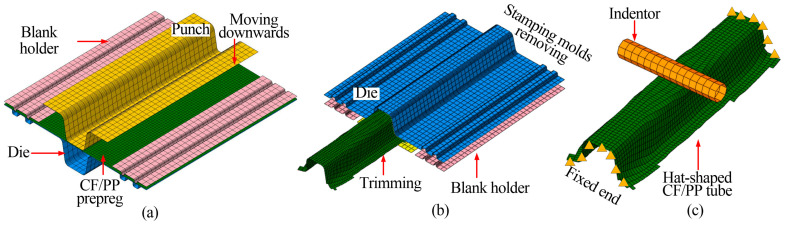
The stamping–bending coupled finite element model of the hat-shaped CF/PP tube: (**a**) the stamping finite element model; (**b**) the molds removing and material trimming finite element model; and (**c**) the bending finite element model.

The stamping–bending coupled finite element model of the CF/PP structure has been developed using ABAQUS, as illustrated in Figure 5. Figure 5a depicts the stamping finite element model, which comprises a punch, a blank holder, a die and the CF/PP prepreg. The punch, the die and the blank holder are treated as rigid components and are discretized using shell elements with dimensions of 10 mm × 10 mm. In contrast, the six–layer CF/PP prepregs are meshed with single-layer shell elements of 5 mm × 5 mm to enhance computational efficiency under the assumption that there is no slippage between adjacent prepregs. The non-orthogonal deformation behavior of the CF/PP prepreg in the stamping process is characterized by the normalized shear force–shear strain curve, as depicted in Figure 1a. Figure 5b illustrates the molds removing finite element model, wherein the preformed CF/PP tube is trimmed by deleting elements that extend beyond the designated target area, and the stamping molds are quickly removed from the preformed sample. Concurrently, the mechanical properties of the preformed CF/PP sample are updated in accordance with the current fiber yarn angles. This study posits that the mechanical properties of materials with fiber angles between 45° to 52° and 52° to 57° are the same as the (+45°/−45°) and (+52°/−52°) laminates, respectively; and the material properties of samples with fiber angles exceeding 57° are analogous to those of the (+57°/−57°) laminate, as summarized in Table 1. Figure 5c presents the bending finite element model of the hat-shaped CF/PP tube, where the indentor is considered a rigid component and is meshed with shell elements of 10 mm × 10 mm. During the bending process, both the right and left ends of the CF/PP tube are fully constrained with six degrees of freedom.

**Figure 6:**
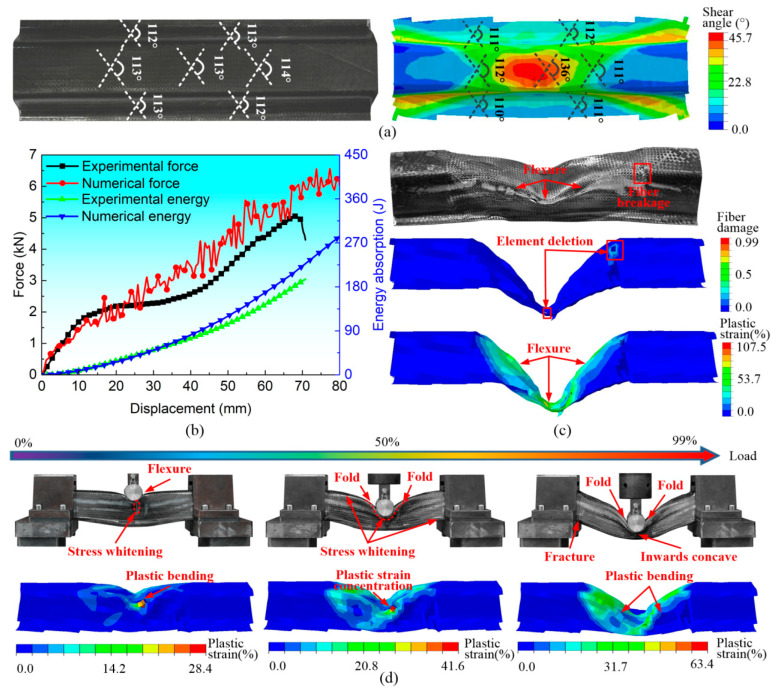
Comparisons in forming and bending performances of the hat-shaped CF/PP specimen between the simulation and experiment results: (**a**) typical fiber angle variations; (**b**) force-/energy–displacement curves; (**c**) bending damage modes; and (**d**) bending deformation histories.

Based on the stamping–bending coupled FEM, the shear characteristics and bending responses of the hat-shaped CF/PP tube were predicted and compared with experimental results, as shown in Figure 6. In Figure 6a, the predicted shear angle distributions and values exhibited some discrepancies when compared to the experimental data. The shear concentration is observed to be significantly higher in the central region of the CF/PP tube, approximately 20% greater than the experimental results. In comparison, the shear angle values on the external surface of the physical CF/PP hollow were found to be relatively consistent. The predicted force-/energy–displacement curves and damaged positions, as well as failure patterns, demonstrated a strong correlation with the experimental results, as shown in Figure 6b,c. It is noteworthy that the predicted peak force and the related failure displacement of predictions were slightly greater than those observed in the experimental data. Nevertheless, the three–point bending behaviors of the CF/PP tube predicted by coupled FEM were consistent with physical tests. It was observed that the shear plastic strain initially concentrated in the central region of the specimen, subsequently expanding from the center to the lateral areas as the indenter continued its downward movement. Ultimately, the maximum shear plastic strain reached approximately 63.4%, and the failure elements at the clamping end were removed, as shown in Figure 6d.

A notable discrepancy has been observed between the simulation and experimental results regarding maximum shear angles. This discrepancy can be attributed to the actual stamping temperature being marginally lower than the targeted 220 °C due to the heat loss during the mold closure process since the hot pressing machine lacks an insulation device, as shown in Figure 1a. The reduction in actual temperature resulted in inadequate melting of polypropylene, which in turn increased the shear stiffness of the CF/PP prepreg. Consequently, the maximum shear angle of the physical specimen is smaller than the numerical result. Furthermore, the larger shear angles further resulted in significant variations in mechanical properties of the preformed CF/PP tube since the strength parameters and failure strains of non-orthogonal CF/PP laminates are closely linked to the fiber yarn angles, as shown in Figure 1c,d. On the other hand, the real-life six–layer CF/PP prepregs were modeled by the single-layer shell elements in the stamping–bending coupled finite element model, and this simplified modeling method omits the interlayer interactions of CF/PP tubes. Moreover, the current study is limited to conducting bias-extension tests on only three non-orthogonal CF/PP laminates due to constraints related to experimental costs and testing conditions and posits that the mechanical properties of materials with fiber angles ranging from 45° to 52° and from 52° to 57° are equivalent to those of the (+45°/−45°) and (+52°/−52°) laminates, respectively; however, the fiber angles exceeding 57° are considered analogous to those of the (+57°/−57°) laminate. This assumption also contributed to the observed differences in the peak force between the physical test and numerical prediction. Although there are some discrepancies observed in the maximum shear angle and peak force between the numerical and experimental results, the present multi-step analysis method still achieved a stamping–bending coupled simulation with tolerable errors. The coupled finite element model can be utilized for conducting parametric studies and optimization designs of CF/PP structures.

## 3. Influence of Stamping Condition on Bending Performance

### 3.1. Cross-Sectional Shape (CSS)

**Figure 7:**
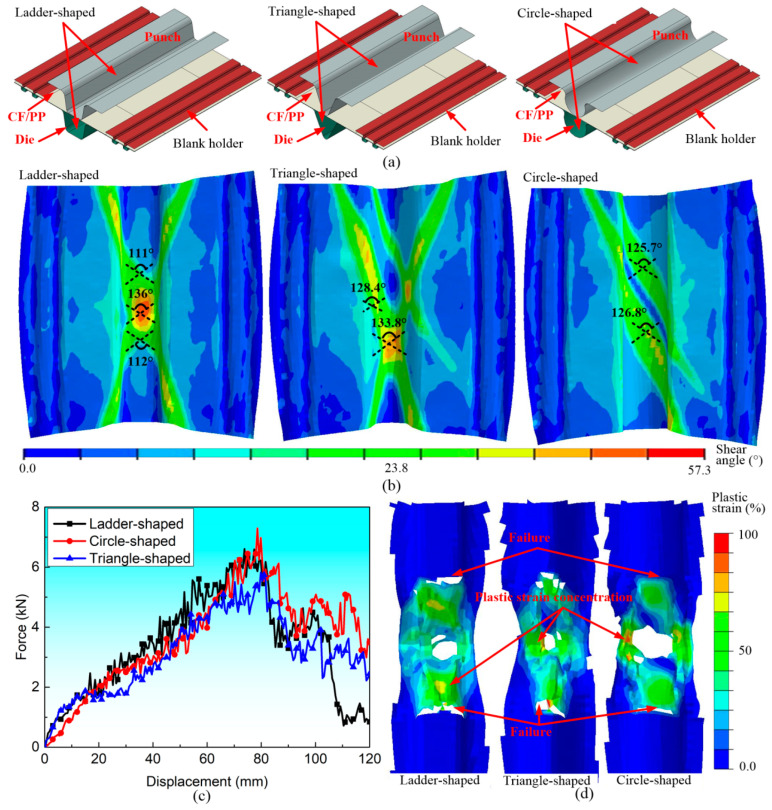
The influences of the cross-sectional shape on the forming and bending characteristics of the CF/PP specimens: (**a**) the diagrams of stamping molds with three different cross-sectional shapes; (**b**) the fiber yarn shear angle distributions after the stamping process; (**c**) the bending force–displacement curves; and (**d**) the ultimate damage modes and plastic deformations.

This section will examine the influence of the cross-sectional shape (*CSS*) of the CF/PP hollow structures on the characteristics of shear angle and bending performance. Figure 7a illustrates three different FEMs featuring ladder-/circle-/triangle-shaped molds, respectively. The cross-sectional lengths of different molds kept constant to uniform mass. The distribution of shear angle exhibited notable differences among the three specimens. Specifically, the shear angle concentration ribbons of both ladder-shaped and triangle-shaped samples displayed crossed configurations, which are quite different from the circle-shaped specimen, as shown in Figure 7b. However, the TPB force–displacement curves and the failure modes of these three specimens are similar to each other, as shown in Figure 7c,d.

### 3.2. Blank Holding Ring Shape (BHRS)

**Figure 8:**
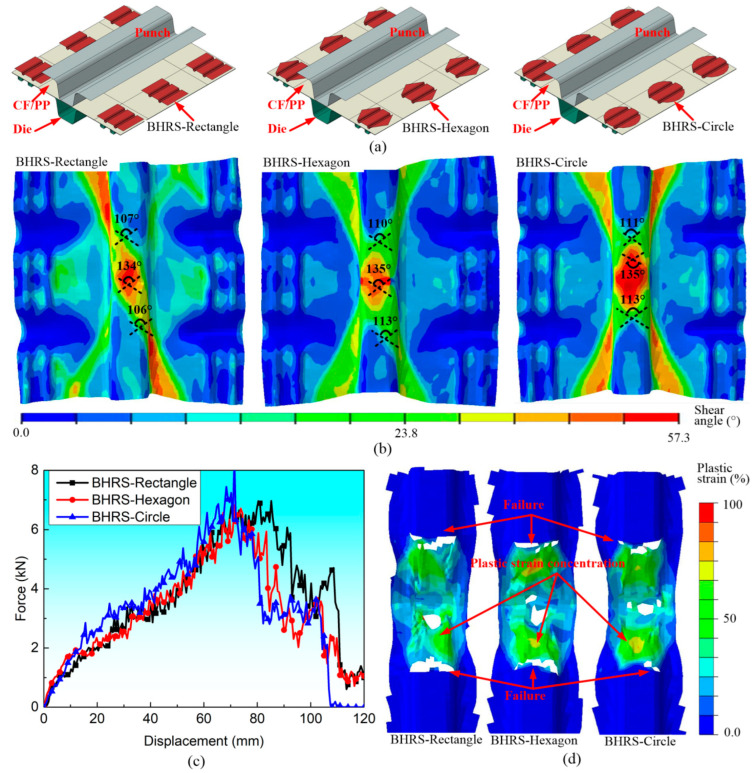
The influences of the blank holding ring shapes on the forming and bending characteristics of the CF/PP specimens: (**a**) the diagrams of stamping molds with three different blank holding ring shapes; (**b**) the fiber yarn shear angle distributions after the stamping process; (**c**) the bending force–displacement curves; and (**d**) the ultimate damage modes and plastic deformations.

Figure 8 presents a comparative analysis of the effects of three distinct blank holding ring shapes (*BHRS*) on the forming characteristics and bending responses of CF/PP specimens. These three bland holders are depicted in Figure 8a, namely *BHRS*-rectangle, *BHRS*-hexagon and *BHRS*-circle, respectively, all of which possess equivalent surface areas. From Figure 8b, it can be seen that the shear angle distribution characteristics and the maximum shear angle values for these specimens exhibit notable similarities. Furthermore, the TPB force–displacement curves, the damaged patterns and the plastic strain were also close to each other, as shown in Figure 8c,d.

### 3.3. Blank Holding Ring Length (BHRL)

**Figure 9:**
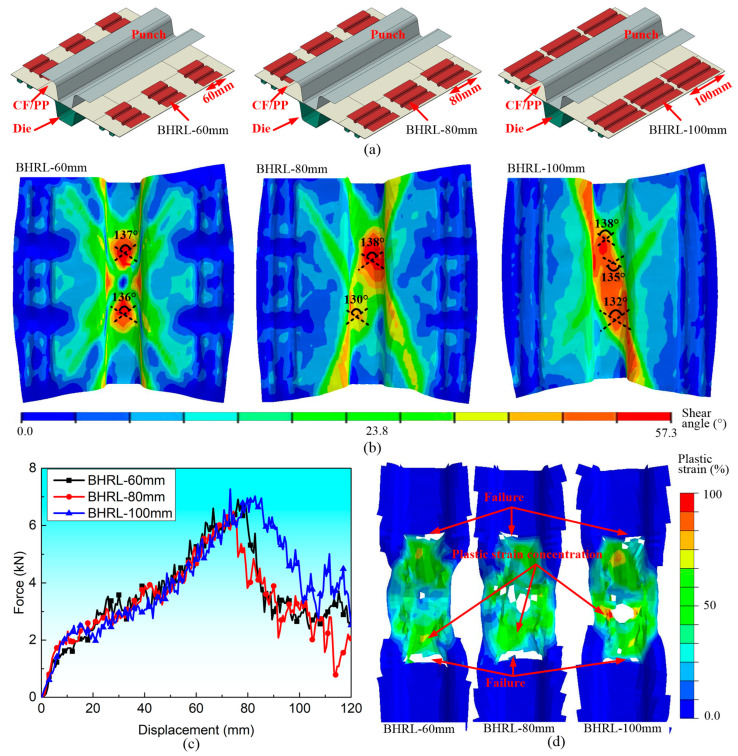
The influences of the blank holding ring lengths on the forming and bending characteristics of the CF/PP specimens: (**a**) the diagrams of stamping molds with three different blank holding ring lengths; (**b**) the fiber yarn shear angle distributions after the stamping process; (**c**) the bending force–displacement curves; and (**d**) the ultimate damage modes and plastic deformations.

Figure 9a shows three different coupled FEMs characterized by various blank holding ring lengths (*BHRL*). The shear angle began to exhibit an asymmetric distribution as the BHRL increased from 60 mm to 100 mm. However, the maximum shear angles remained relatively comparable, as depicted in Figure 9b. Prior to the onset of failure, the bending forces across all specimens were similar, but the bending force of the greatest *BHRL* was significantly higher than the other two samples thereafter, as shown in Figure 9c. Nonetheless, the damage modes and the shear plastic strain among the three specimens were found to be analogous, as shown in Figure 9d.

**Figure 10:**
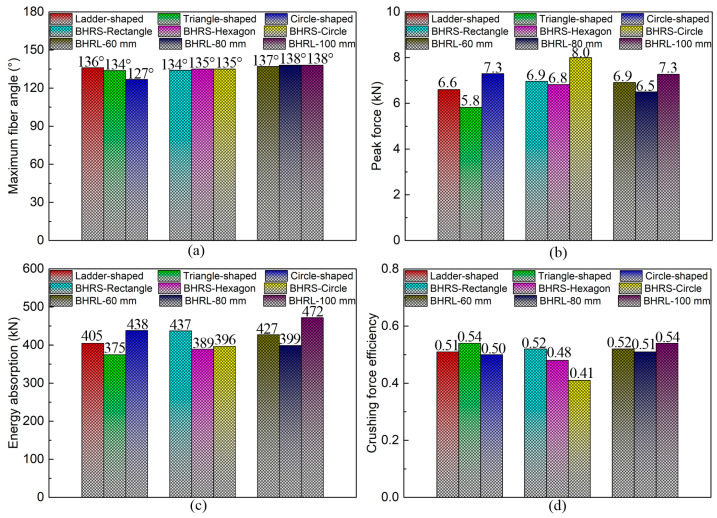
Comparisons in the forming and bending performance indicators of the CF/PP specimens: (**a**) the maximum fiber angle after the stamping process; (**b**) the peak force indicator; (**c**) the energy absorption indicator; and (**d**) the crushing force efficiency indicator.

Figure 10 shows a comparative analysis of the forming and bending performance indicators for the CF/PP specimens. It is evident that both the blank holding ring size (*BHRS*) and blank holding ring length (*BHRL*) variables exert minimal influence on the maximum fiber angle forming indicators, while the cross-sectional shape (*CSS*) variable caused the decrease in maximum fiber angle (i.e., the mold cross-sectional shape varied from the ladder to the circle configuration), as shown in Figure 10a. For the peak force (*PF*), energy absorption (*EA*) and crushing force efficiency (*CFE*) bending performance indicators, all *CSS*, *BHRS* and *BHRL* variables demonstrate significant effects on the CF/PP specimens, as shown in Figure 10b,c. Notably, the *EA* and *CFE* indicators of the CF/PP specimens can be improved by increasing the length of the blank holding ring, and the CF/PP specimen constrained by the blank holding ring with 100 mm in length exhibits the highest levels of *EA* and *CFE*.

## 4. Multi-Objective Discrete Optimization Design

### 4.1. Optimization Problem Definition

The parametric analyses indicate that blank holding ring length (*BHRL*) can affect the forming and bending performance to a certain degree. Previous studies have also demonstrated that the blank holding ring force (*BHRF*) can significantly impact the forming behaviors of woven fabrics [10]. Moreover, the thickness of the composite specimen is a critical factor in the bending process [45]. Thus, the *BHRF*, *BHRL* and the thickness (*T*) were identified as three design variables, while the energy absorption (*EA*) and the material mass (*M*) were taken as optimization objectives. There are 10 different thickness variables. A total of ten distinct variables for *T*, *BHRF* and *BHRL* are presented in Table 2.

On the other hand, the bias-extension test conducted on the single CF/PP prepreg at a temperature the 220 °C revealed that the maximum shear angle was approximately 0.9 radians. To ensure the formability of the structural optimization design, it is imperative that the maximum shear angle during the stamping process is treated as a constraint, which is not allowed to exceed the limit shear angle (i.e., 0.9 radians). Therefore, the mathematical formulation of the optimization problem can be expressed as follows:

(12) $$\left\{\begin{array}{l} F\left(x\right)=\max\left\{EA\left(T,BHRF,BHRL\right),-W\left(T,BHRF,BHRL\right)\right\}\\ s.t\left\{\begin{array}{l} \theta_{max}\left(x\right)\leq 0.9\\ \begin{array}{l} T\in \left(T1,T2,T3,T4,T5,T6,T7,T8,T9,T10\right)\\ BHRF\in \left(F1,F2,F3,F4,F5,F6,F7,F8,F9,F10\right)\\ BHRL\in \left(L1,L2,L3,L4,L5,L6,L7,L8,L9,L10\right) \end{array} \end{array}\right. \end{array}\right.$$

In this study, a penalty function was employed to address the constraint issue, whereby the object incurs a penalty if the maximum shear angle ($\theta_{max}$) exceeds the specified constraint condition. The formulation of the penalty function ($\theta_{max,new}\left(x\right)$) is provided in references [46,47]:

(13) $$\theta_{max,new}\left(x\right)=P_{1}\times max\left[0,(\theta_{max}-0.9)\right]$$

where $P_{1}$ is the penalized factor as reported in [46,47].

Consequently, the mathematical formulation of the optimization problem can be restructured as follows:

(14) $$\left\{\begin{array}{l} \begin{array}{l} f_{1}(x)=\max\left\{EA\left(T,BHRF,BHRL\right)+\theta_{max,new}\left(T,BHRF,BHRL\right)\right\}\\ f_{2}(x)=\max\left\{-W\left(T,BHRF,BHRL\right)+\theta_{max,new}\left(T,BHRF,BHRL\right)\right\} \end{array}\\ s.t\left\{\begin{array}{l} T\in \left(T1,T2,T3,T4,T5,T6,T7,T8,T9,T10\right)\\ BHRF\in \left(F1,F2,F3,F4,F5,F6,F7,F8,F9,F10\right)\\ BHRL\in \left(L1,L2,L3,L4,L5,L6,L7,L8,L9,L10\right) \end{array}\right. \end{array}\right.$$

where $f_{1}(x)$ and $f_{2}(x)$ are the penalized objective functions, respectively.

**Figure 11:**
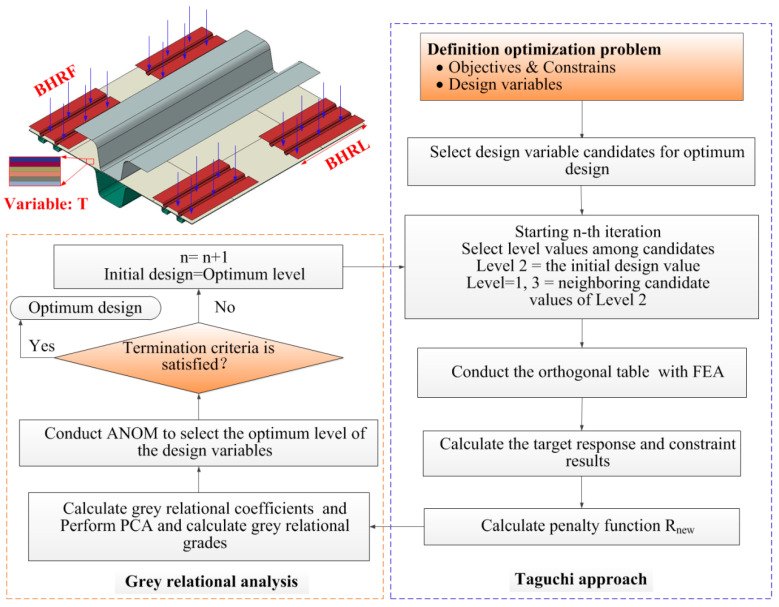
The multi-objective discrete optimization flowchart of CF/PP hat-shaped specimen accounting for the stamping process effects, in which the Taguchi approach was employed to deal with the discrete variables, and the gray relational analysis method was adopted to transform the constrained multi-objective problems into the unconstrained single-objective problems.

The optimization problem described above involves several discrete design variables, which presents challenges for the optimization process. Previous studies have demonstrated that such problems can be effectively and reliably addressed through a collaborative integration of the Taguchi method, gray relational analysis (GRA) and the analysis of mean (ANOM) method [48,49,50]. The optimization flowchart is depicted in Figure 11. The Taguchi approach is employed to conduct the design of experiments (DOE) during each iteration. Subsequently, the penalized objectives are analyzed using the GRA procedure to identify the optimal levels of each design variable and to establish the DOE for the subsequent iteration. The optimization process concludes when the gray relational degree (GRD) remains constant over five consecutive iterations or when the optimal levels of all design variables simultaneously shift to the second level.

### 4.2. Optimization Design Result

Table 3 lists the orthogonal array for the first iteration, in which the combination of (*T6*, *F6* and *L6*) was determined as the second level of the first iteration, and the adjacent variables of these three samples were defined as the first and third levels, respectively. Subsequently, the penalized objective responses of the nine design schemes of the 1st iteration were computed utilizing the coupled FEMs and Equations (11) and (12), as detailed in Table 4. Furthermore, the aforementioned penalized objective values were subjected to a gray relational analysis procedure, and the related gray relational parameters can be calculated, as summarized in Table 5. Then, the analysis of the mean producer for the gray relational degree of these nine samples was conducted, as outlined in Table 6. The results indicated that the first level was for all three variables simultaneously, as presented in Table 7. Thus, the combination of (*T5*, *F5* and *L5*) was identified as the second level for the second iteration procedure.

**Figure 12:**
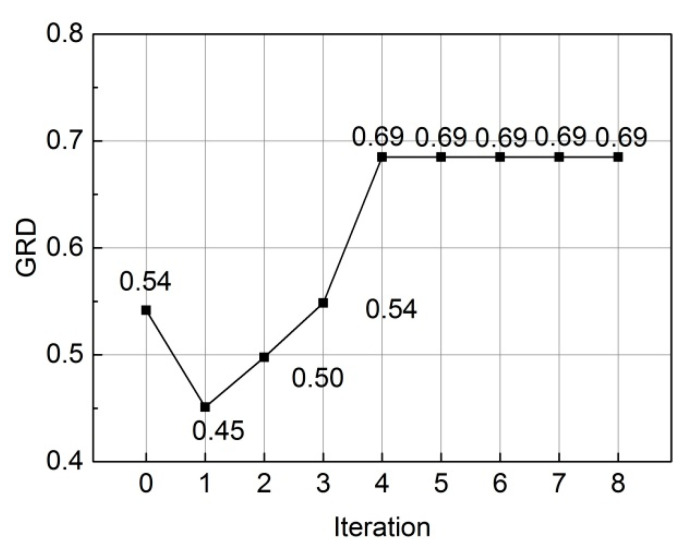
Iteration history of gray relational degree.

The optimum process was characterized by a series of iterations, with the results from the second to fifth iterations summarized in Table A1 through Table A20 (second iteration: Table A1, Table A2, Table A3, Table A4 and Table A5; third iteration: Table A6, Table A7, Table A8, Table A9 and Table A10; fourth iteration: Table A11, Table A12, Table A13, Table A14 and Table A15; fifth iteration: Table A16, Table A17, Table A18, Table A19 and Table A20), as detailed in the Appendix A. It was worth noticing that the optimum levels of three design variables simultaneously transitioned to the second levels, leading to the termination of the optimum process upon the fulfillment of the convergence criterion. Figure 12 illustrates a comparison of the gray relational degree values across all iterations. Consequently, the optimum design was determined as the combination of (*T2*, *F5* and *L5*). Table 8 further provides a comparative analysis of the mass (M), maximum shear angle ($\theta_{max}$), energy absorption (EA) and special energy absorption (SEA) metrics between the baseline design and optimum design. Although the EA and $\theta_{max}$ were found to be comparable, however, the SEA exhibited an improvement of 17.5% whereas the M was reduced by 14.3%. This indicates a significant enhancement in bending performance without the addition of extra mass, in accordance with the constraints imposed by the stamping process.

## 5. Conclusions

This research initially employed the hot stamping technology to fabricate a hat-shaped CF/PP hollow structure characterized by a non-orthogonal fiber configuration. Subsequently, the three-point bending performance of the trimmed CF/PP hollow structure was investigated through experimental methodologies. Based on the experimental findings, the forming-performance integrated finite element model (FEM) was developed using ABAQUS/Explicit, wherein a multi-step analysis approach was effectively utilized to facilitate the integrated procedure. Furthermore, the parametric study and the integrated optimization design focusing on the bending performance of CF/PP hollow structures were conducted. The following conclusions can be drawn from this study:

(1) The orthogonal configuration of the fabric in CF/PP prepreg was altered to the non-orthogonal configuration due to the constraints imposed by the blank holder. In off-axis tensile tests, laminates with a larger non-orthogonal fiber yarn angle exhibited an increased failure strain, albeit with a reduced failure strength. Furthermore, in the three-point bending test, the non-orthogonal CF/PP hollow specimen exhibited significant shear plastic deformations, with the maximum plastic strain reaching approximately 63.4% at the failure point.
(2) The influences of the blank holding ring shape (*BHRS*) on the shear angle characteristics and bending responses of the CF/PP hollow specimens were not significant. In contrast, the cross-sectional shape (*CSS*) and blank holding ring length (*BHRL*) displayed significant variations in shear angle distributions.
(3) The multi-objective discrete optimization for the three-point bending performance of CF/PP structures accounting for the influences of the stamping process was successfully implemented. The optimized design achieved an SEA improvement of 17.5% while simultaneously reducing mass by 14%, and the maximum shear angle only increased by 1.4% compared to the baseline design, remaining below the specified limit shear angle.

## Figures and Tables

**Table 1:** Summary of mechanical properties of CF/PP laminates.

Samples	Strength (Mpa)	Modulus (Mpa)	Failure Strain (%)	Yield Strength (Mpa)
(+45°/−45°)_6_	41	550	79.1	11.0
(+52°/−52°)_6_	22	475	98.5	9.5
(+57°/−57°)_6_	9	435	105.6	6.5

**Table 2:** Summary of design schemes of design variables and related descriptions.

*T*	Description	*BHRF*	Description	*BHRL*	Description
*T1*	5-ply	*F1*	700 N	*L1*	85 mm
*T2*	6-ply	*F2*	900 N	*L2*	95 mm
*T3*	7-ply	*F3*	1100 N	*L3*	105 mm
*T4*	8-ply	*F4*	1300 N	*L4*	115 mm
*T5*	9-ply	*F5*	1500 N	*L5*	125 mm
*T6*	10-ply	*F6*	1700 N	*L6*	135 mm
*T7*	11-ply	*F7*	1900 N	*L7*	145 mm
*T8*	12-ply	*F8*	2100 N	*L8*	155 mm
*T9*	13-ply	*F9*	2300 N	*L9*	165 mm
*T10*	14-ply	*F10*	2500 N	*L10*	175 mm

**Table 3:** The orthogonal array of the first iteration.

Design Variables	Level		
	1	2	3
T	*T5*	*T6*	*T7*
BHRF	*F5*	*F6*	*F7*
BHRL	*L5*	*L6*	*L7*

**Table 4:** Penalized objectives of the first iteration.

No.	Design Variables			Objective Functions	
	T	BHRF	BHRL	$f_{1}(x)$	$f_{2}(x)$
1	*T5*	*F5*	*L5*	0.544	−0.101
2	*T5*	*F6*	*L6*	0.529	−0.101
3	*T5*	*F7*	*L7*	0.615	−0.103
4	*T6*	*F5*	*L7*	0.795	−0.112
5	*T6*	*F6*	*L6*	0.615	−0.115
6	*T6*	*F7*	*L5*	0.737	−0.112
7	*T7*	*F5*	*L6*	1.049	−0.123
8	*T7*	*F6*	*L7*	1.034	−0.123
9	*T7*	*F7*	*L5*	1.014	−0.123

**Table 5:** Gray relational analysis responses of the first iteration.

No.			Sequence after Normalizing		Gray Relational Coefficient		GRD
	$f_{1}(x)$	$f_{2}(x)$	$f_{1}(x)$	$f_{2}(x)$	$f_{1}(x)$	$f_{2}(x)$	
1	0.544	−0.101	0.029	1.000	0.340	1.000	0.670
2	0.529	−0.101	0.000	1.000	0.333	1.000	0.667
3	0.615	−0.103	0.165	0.911	0.375	0.848	0.612
4	0.795	−0.112	0.511	0.500	0.506	0.500	0.503
5	0.615	−0.115	0.165	0.366	0.375	0.441	0.408
6	0.737	−0.112	0.401	0.500	0.455	0.500	0.477
7	1.049	−0.123	1.000	0.000	1.000	0.333	0.667
8	1.034	−0.123	0.970	0.000	0.944	0.333	0.639
9	1.014	−0.123	0.933	0.000	0.881	0.333	0.607

**Table 6:** Analysis of mean of the gray relational degree of the first iteration.

Design Variables	Levels		
	1	2	3
T	0.643	0.458	0.631
BHRF	0.607	0.565	0.560
BHRL	0.589	0.586	0.556

**Table 7:** Optimum levels of the first iteration.

Design Variables	Level		
	1	2	3
T	*T5*	*T6*	*T7*
BHRF	*F5*	*F6*	*F7*
BHRL	*L5*	*L6*	*L7*

**Note:** The bold levels represent the optimum combination within the current iteration.

**Table 8:** Comparison between initial baseline design and optimum design.

Comparison	Scheme	M (g)	EA (J)	SEA (J/g)	$\theta_{max}$ (rad)
**Baseline design**	T3F4L4	78.4	349.63	4.46	0.84
**Optimum design**	T2F5L5	67.2	352.18	5.24	0.85
**Improvement**		−14.3%	+0.7%	+17.5%	+1.2%

## Data Availability

Data is contained within the article.

## Appendix A

Table A1 lists the orthogonal array for the second iteration, in which the combination of (*T5*, *F5* and *L5*) was identified as the second level, while the adjacent variables were designated as the first and third levels, respectively. Subsequently, the penalized objective responses for nine design schemes of the second iteration were calculated, as summarized in Table A2. Furthermore, the above penalized objective values were subjected to the gray relational analysis procedure, and the related gray relational parameters can be calculated, as summarized in Table A3. Then, the analysis of the mean producer for the gray relational degree of these nine samples was conducted, as shown in Table A4. The analysis revealed that the combination of (*T4*, *F5* and *L5*) was the optimum level for these three variables simultaneously, as listed in Table A5, which was identified as the second level of the third iteration procedure.

**Table A1:** The orthogonal array of the second iteration procedure.

Design Variables	Level		
	1	2	3
T	*T4*	*T5*	*T6*
BHFR	*F4*	*F5*	*F6*
BHRL	*L4*	*L5*	*L6*

**Table A2:** Penalized objectives of the second iteration procedure.

No.	Design Variables			Objective Functions	
	T	BHRF	BHRL	$f_{1}(x)$	$f_{2}(x)$
1	*T4*	*F4*	*L4*	0.528	−0.090
2	*T4*	*F5*	*L5*	0.529	−0.090
3	*T4*	*F6*	*L6*	0.500	−0.090
4	*T5*	*F4*	*L6*	0.570	−0.101
5	*T5*	*F5*	*L5*	0.544	−0.101
6	*T5*	*F6*	*L4*	0.513	−0.101
7	*T6*	*F4*	*L5*	0.691	−0.112
8	*T6*	*F5*	*L6*	0.700	−0.112
9	*T6*	*F6*	*L4*	0.691	−0.112

**Table A3:** Gray relational analysis responses of the second iteration procedure.

No.			Sequence after Normalizing		Gray Relational Coefficient		GRD
	$f_{1}(x)$	$f_{2}(x)$	$f_{1}(x)$	$f_{2}(x)$	$f_{1}(x)$	$f_{2}(x)$	
1	0.528	−0.090	0.140	1.000	0.368	1.000	0.684
2	0.529	−0.090	0.146	1.000	0.369	1.000	0.685
3	0.500	−0.090	0.000	1.000	0.333	1.000	0.667
4	0.570	−0.101	0.351	0.500	0.435	0.500	0.468
5	0.544	−0.101	0.222	0.500	0.391	0.500	0.446
6	0.513	−0.101	0.068	0.500	0.349	0.500	0.425
7	0.691	−0.112	0.956	0.000	0.920	0.333	0.626
8	0.700	−0.112	1.000	0.000	1.000	0.333	0.667
9	0.691	−0.112	0.956	0.000	0.920	0.333	0.627

**Table A4:** Analysis of mean of the gray relational degree of the second iteration procedure.

Design Variables	Levels		
	1	2	3
T	0.672	0.441	0.633
BHRF	0.587	0.593	0.567
BHRL	0.586	0.587	0.574

**Table A5:** Optimum levels of the second iteration procedure.

Design Variables	Level		
	1	2	3
T	*T4*	*T5*	*T6*
BHRF	*F4*	*F5*	*F6*
BHRL	*L4*	*L5*	*L6*

**Note:** The bold levels represent the optimum combination within the current iteration.

The combination of (*T4*, *F5* and *L5*) was determined as the second level of the third iteration, as summarized in Table A6. Subsequently, the penalized objective responses of nine design schemes of the third iteration were calculated, as summarized in Table A7. Furthermore, Table A8 exhibits the related gray relational parameters of these nine penalized objective values. Then, the analysis of the mean results of these nine samples was listed in Table A9, and the optimum combination of (*T3*, *F5* and *L5*) was identified as the second level of the 4th iteration procedure, as shown in Table A10.

**Table A6:** The orthogonal array of the third iteration procedure.

Design Variables	Level		
	1	2	3
T	*T3*	*T4*	*T5*
BHRF	*F4*	*F5*	*F6*
BHRL	*L4*	*L5*	*L6*

**Table A7:** Penalized objectives of the third iteration procedure.

No.	Design Variables			Objective Functions	
	T	BHRF	BHRL	$f_{1}(x)$	$f_{2}(x)$
1	*T3*	*F4*	*L4*	0.350	−0.078
2	*T3*	*F5*	*L5*	0.388	−0.078
3	*T3*	*F6*	*L6*	0.385	−0.078
4	*T4*	*F4*	*L6*	0.534	−0.090
5	*T4*	*F5*	*L5*	0.529	−0.090
6	*T4*	*F6*	*L4*	0.482	−0.090
7	*T5*	*F4*	*L5*	0.474	−0.101
8	*T5*	*F5*	*L6*	0.550	−0.101
9	*T5*	*F6*	*L4*	0.513	−0.101

**Table A8:** Gray relational analysis responses of the third iteration procedure.

No.			The Sequence after Normalizing		Gray Relational Coefficient		GRD
	*f*_1_(x)	*f*_2_(x)	*f*_1_(x)	*f*_2_(x)	*f*_1_(x)	*f*_2_(x)	
1	0.350	−0.078	0.000	1.000	0.333	1.000	0.667
2	0.388	−0.078	0.193	1.000	0.382	1.000	0.691
3	0.385	−0.078	0.174	1.000	0.377	1.000	0.689
4	0.534	−0.090	0.919	0.500	0.861	0.500	0.680
5	0.529	−0.090	0.894	0.500	0.826	0.500	0.663
6	0.482	−0.090	0.662	0.500	0.597	0.500	0.548
7	0.474	−0.101	0.618	0.000	0.567	0.333	0.450
8	0.550	−0.101	1.000	0.000	1.000	0.333	0.667
9	0.513	−0.101	0.817	0.000	0.732	0.333	0.533

**Table A9:** Analysis of mean of the gray relational degree of the third iteration procedure.

Design Variables	Levels		
	1	2	3
T	0.675	0.624	0.544
BHRF	0.593	0.667	0.584
BHRL	0.621	0.628	0.595

**Table A10:** Optimum levels of the third iteration procedure.

Design Variables	Level		
	1	2	3
T	*T3*	*T4*	*T5*
BHRF	*F4*	*F5*	*F6*
BHRL	*L4*	*L5*	*L6*

**Note:** The bold levels represent the optimum combination within the current iteration.

The combination of (*T3*, *F5* and *L5*) was determined as the second level of the fourth iteration, as summarized in Table A11. Subsequently, the penalized objective responses of nine design schemes of the fourth iteration were calculated, as summarized in Table A12. Furthermore, Table A13 exhibits the related gray relational parameters of these nine penalized objective values. Then, the analysis of the mean results of these nine samples was listed in Table A14, and the optimum combination of (*T2*, *F5* and *L5*) was determined to be the second level of the fifth iteration procedure, as shown in Table A15.

**Table A11:** The orthogonal array of the fourth iteration procedure.

Design Variables	Level		
	1	2	3
T	*T2*	*T3*	*T4*
BHRF	*F4*	*F5*	*F6*
BHRL	*L4*	*L5*	*L6*

**Table A12:** Penalized objectives of the fourth iteration procedure.

No.	Design Variables			Objective Functions	
	T	BHRF	BHRL	$f_{1}(x)$	$f_{2}(x)$
1	*T2*	*F4*	*L4*	0.314	−0.067
2	*T2*	*F5*	*L5*	0.352	−0.067
3	*T2*	*F6*	*L6*	0.344	−0.082
4	*T3*	*F4*	*L6*	0.418	−0.078
5	*T3*	*F5*	*L5*	0.388	−0.078
6	*T3*	*F6*	*L4*	0.396	−0.082
7	*T4*	*F4*	*L5*	0.487	−0.090
8	*T4*	*F5*	*L6*	0.497	−0.090
9	*T4*	*F6*	*L4*	0.482	−0.090

**Table A13:** Analysis of mean responses of the fourth iteration procedure.

No.			The Sequence after Normalizing		Gray Relational Coefficient		GRD
	*f*_1_(x)	*f*_2_(x)	*f*_1_(x)	*f*_2_(x)	*f*_1_(x)	*f*_2_(x)	
1	0.314	−0.067	0.000	1.000	0.333	1.000	0.667
2	0.352	−0.067	0.210	1.000	0.388	1.000	0.694
3	0.344	−0.082	0.164	0.330	0.374	0.427	0.401
4	0.418	−0.078	0.571	0.500	0.538	0.500	0.519
5	0.388	−0.078	0.407	0.500	0.457	0.500	0.479
6	0.396	−0.082	0.451	0.321	0.477	0.424	0.450
7	0.487	−0.090	0.947	0.000	0.903	0.333	0.618
8	0.497	−0.090	1.000	0.000	1.000	0.333	0.667
9	0.482	−0.090	0.920	0.000	0.862	0.333	0.598

**Table A14:** Analysis of mean of the gray relational degree of the fourth iteration procedure.

Design Variables	Levels		
	1	2	3
T	0.581	0.478	0.621
BHRF	0.595	0.607	0.478
BHRL	0.589	0.597	0.494

**Table A15:** Optimum levels of the fourth iteration procedure.

Design Variables	Level		
	1	2	3
T	*T2*	*T3*	*T4*
BHRF	*F4*	*F5*	*F6*
BHRL	*L4*	*L5*	*L6*

**Note:** The bold levels represent the optimum combination within the current iteration.

The combination of (*T2*, *F5* and *L5*) was determined as the second level of the fifth iteration, as summarized in Table A16. Subsequently, the penalized objective responses of nine design schemes of the fifth iteration were calculated, as summarized in Table A17. Furthermore, Table A18 exhibits the related gray relational parameters of these nine penalized objective values. Then, the analysis of the mean results of these nine samples was listed in Table A19. Finally, the optimum combination was determined as (*T2*, *F5* and *L5*), which was the same as the initial combination of the second level of the fifth iteration, and the combination of (*T2*, *F5* and *L5*) was selected as the ultimate optimum design scheme, as shown in Table A20.

**Table A16:** The orthogonal array of the fifth iteration procedure.

Design Variables	Level		
	1	2	3
T	*T1*	*T2*	*T3*
BHRF	*F4*	*F5*	*F6*
BHRL	*L4*	*L5*	*L6*

**Table A17:** Penalized objectives of the fifth iteration procedure.

No.	Design Variables			Objective Functions	
	T	BHRF	BHRL	$f_{1}(x)$	$f_{2}(x)$
1	*T1*	*F4*	*L4*	0.276	−0.069
2	*T1*	*F5*	*L5*	0.304	−0.071
3	*T1*	*F6*	*L6*	0.230	−0.076
4	*T2*	*F4*	*L6*	0.307	−0.067
5	*T2*	*F5*	*L5*	0.352	−0.067
6	*T2*	*F6*	*L4*	0.306	−0.078
7	*T3*	*F4*	*L5*	0.389	−0.078
8	*T3*	*F5*	*L6*	0.410	−0.078
9	*T3*	*F6*	*L4*	0.396	−0.082

**Table A18:** Gray relational analysis responses of the fifth iteration procedure.

No.			The Sequence after Normalizing		Gray Relational Coefficient		GRD
	*f*_1_(x)	*f*_2_(x)	*f*_1_(x)	*f*_2_(x)	*f*_1_(x)	*f*_2_(x)	
1	0.276	−0.069	0.257	0.862	0.402	0.784	0.593
2	0.304	−0.071	0.410	0.783	0.459	0.697	0.578
3	0.230	−0.076	0.000	0.421	0.333	0.463	0.398
4	0.307	−0.067	0.428	1.000	0.466	1.000	0.733
5	0.352	−0.067	0.678	1.000	0.609	1.000	0.804
6	0.306	−0.078	0.424	0.270	0.465	0.406	0.436
7	0.389	−0.078	0.884	0.263	0.812	0.404	0.608
8	0.410	−0.078	1.000	0.263	1.000	0.404	0.702
9	0.396	−0.082	0.924	0.000	0.868	0.333	0.601

**Table A19:** Analysis of mean of the gray relational degree of the fifth iteration procedure.

Design Variables	Levels		
	1	2	3
T	0.518	0.651	0.631
BHRF	0.638	0.688	0.473
BHRL	0.571	0.631	0.597

**Table A20:** Optimum levels of the fifth iteration procedure.

Design Variables	Level		
	1	2	3
T	*T1*	*T2*	*T3*
BHRF	*F4*	*F5*	*F6*
BHRL	*L4*	*L5*	*L6*

**Note:** The bold levels represent the optimum combination within the current iteration.
